# Distributed encoding of spatial and object categories in primate hippocampal microcircuits

**DOI:** 10.3389/fnins.2015.00317

**Published:** 2015-10-06

**Authors:** Ioan Opris, Lucas M. Santos, Greg A. Gerhardt, Dong Song, Theodore W. Berger, Robert E. Hampson, Sam A. Deadwyler

**Affiliations:** ^1^Department of Physiology and Pharmacology, Wake Forest University School of MedicineWinston-Salem, NC, USA; ^2^Department of Anatomy and Neurobiology, University of KentuckyLexington, KY, USA; ^3^Department of Biomedical Engineering, University of Southern CaliforniaLos Angeles, CA, USA

**Keywords:** hippocampus, CA1, CA3, spatial representation, numeric categorization, patterned microstimulation, cognitive function, rhesus macaque monkey

## Abstract

The primate hippocampus plays critical roles in the encoding, representation, categorization and retrieval of cognitive information. Such cognitive abilities may use the transformational input-output properties of hippocampal laminar microcircuitry to generate spatial representations and to categorize features of objects, images, and their numeric characteristics. Four nonhuman primates were trained in a delayed-match-to-sample (DMS) task while multi-neuron activity was simultaneously recorded from the CA1 and CA3 hippocampal cell fields. The results show differential encoding of spatial location and categorization of images presented as relevant stimuli in the task. Individual hippocampal cells encoded visual stimuli only on specific types of trials in which retention of either, the Sample image, or the spatial position of the Sample image indicated at the beginning of the trial, was required. Consistent with such encoding, it was shown that patterned microstimulation applied during Sample image presentation facilitated selection of either Sample image spatial locations or types of images, during the Match phase of the task. These findings support the existence of specific codes for spatial and numeric object representations in primate hippocampus which can be applied on differentially signaled trials. Moreover, the transformational properties of hippocampal microcircuitry, together with the patterned microstimulation are supporting the practical importance of this approach for cognitive enhancement and rehabilitation, needed for memory neuroprosthetics.

## Introduction

The mammalian hippocampus located in the temporal lobe of primate brain exhibits interconnected circular neuron fields in which CA1 and CA3 cells form a hierarchical network to process sensory information into memory (Mishkin et al., 1997; Lavenex et al., 2006; Amaral and Lavenex, 2007). The hippocampal formation plays a crucial role in *the encoding* of information during memory formation, as well as in categorization and *retrieval* during memory retention (Hampson et al., 1999a, 2004a, 2013). Disruption of hippocampal processing produces memory deficits in animals (Murray et al., 1998; Hampson et al., 1999b; Zola and Squire, 2001) and humans (Scoville and Milner, 1957; Munoz et al., 2011; Smith et al., 2014). An intriguing question regarding the functional roles of CA1 and CA3 cell fields concerns how spatial and image information is actually processed by cells within these structures (Hampson et al., 1999a, 2004a, 2013; Agarwal et al., 2014). One hypothesis posits that cognitive mechanisms in CA1–CA3 may use the input-output transformational properties of hippocampal laminar microcircuits to generate spatial representations and to categorize images and/or numeric features.

Spatial location of objects is encoded by spatial memory that also represents information about their spatial orientation (O'Keefe and Dostrovsky, 1971; Moser and Moser, 1998; Brun et al., 2002). It has been shown that spatial memories form a cognitive map which represents spatial configuration of the optimal path for navigation between arbitrary pairs of points (O'Keefe and Dostrovsky, 1971; Moser and Moser, 1998; Hampson et al., 1999a; Hirabayashi and Miyashita, 2014). In addition, hippocampal cells appear to also have the ability to categorize information in terms of filtering out a multitude of object features to keep only those that might be used for later recall (Hampson et al., 2004a, 2013). However, unexpectedly, the number of images/objects presented also appear to represent a numeric category (Sagiv and Ward, 2006; Hampson et al., 2013). An important characteristic of such numeric categorization is the encoding of displays with the same number of images irrespective of the types of individual images (Snyder et al., 2012; Pagano and Mazza, 2013).

To address the issue of spatial encoding and numeric/feature categorization, simultaneous recording of CA3 and CA1 cells using multi-electrode arrays (Hampson et al., 2004b) and tetrode recording (Santos et al., 2012) was carried out in four rhesus monkeys performing a delayed-match-to sample (DMS) visual discrimination (Hampson et al., 2004a, 2013) task in which spatial location and the number of images were performance factors. Results show that neurons in the hippocampal microcircuits (Sybirska et al., 2000; Förster et al., 2006; Carr and Frank, 2012; Hirabayashi and Miyashita, 2014) within subfields CA1 and CA3 differentially encode visual stimuli (spatial position or the object/numeric features) presented in the same DMS task. These findings show that hippocampal neurons may have the capacity to respond to numeric categories (i.e., numeric quantity) of task related images, in a manner that facilitates performance under conditions where information content from other categories is excessive. Therefore, our results indicate that processes like: (a) spatial encoding, and (b) numeric categorization, occur in the hippocampus of all nonhuman primates performing a cognitive task.

The transformational input-output properties of hippocampal microcircuitry generating spatial representations and categorizing features of objects, images and scenes are consistent to the ongoing paradigm shift in the understanding of memory, by demonstrating a differential encoding of spatial location and categorization of images in the activity of hippocampal cells. The patterned microstimulation applied during the sample image presentation facilitated selection of either sample image spatial locations or the types of images, support the practical importance of this approach for cognitive enhancement and rehabilitation, especially for memory neuroprosthetics.

## Methods

Four adult male rhesus monkeys (9, 10, 12, 14 kg) were trained on a spatial-object rule-based match-to-sample (DMS) visual discrimination task (Hampson et al., 2004a, 2013) (see Supplemental Information). While the monkeys were engaged in the behavioral task (see Supplemental Information), we recorded single- and multiunit responses in the hippocampal CA3 and CA1 subfields. The great majority of neurons were recorded simultaneously, in separate behavioral sessions with tetrodes (Santos et al., 2012). All surgical and behavioral procedures conformed to the guidelines established by the National Institutes of Health and were approved by the Institutional Animal Care and Use Committee of Wake Forest University. We pooled data from multiunit recordings to construct population responses and used trial-based analysis to study the effect of task conditions and time on the population responses. We developed a unique microstimulation approach based on MIMO model (Hampson et al., 2013) that allowed us to identify performance facilitation in various task contexts (encoding and categorization of trial types). The order of the experiments was: (1) each animal was simultaneously trained in both the object, spatial and both combined elements of the DMS task; (2) neurophysiological recordings in each animal, (3) electrical microstimulation using the MIMO model in each animal. The timeline of the experiments was: (1) animal training to fully master the DMS task required training 5 days per week for 1–2 years;-all animals were trained to a 80–90% correct performance level of the task required for continuing in the study; (2) neurophysiological recordings in the DMS task was performed one to two times per week for a determined period of time to collect the data necessary to test the experimental aim; (3) electrical microstimulation with the MIMO model was performed in each animal one session per week during 1–2 months. Detailed descriptions of methods are provided in the Supplementary Material Section.

### Behavioral paradigm

**Figure 1:**
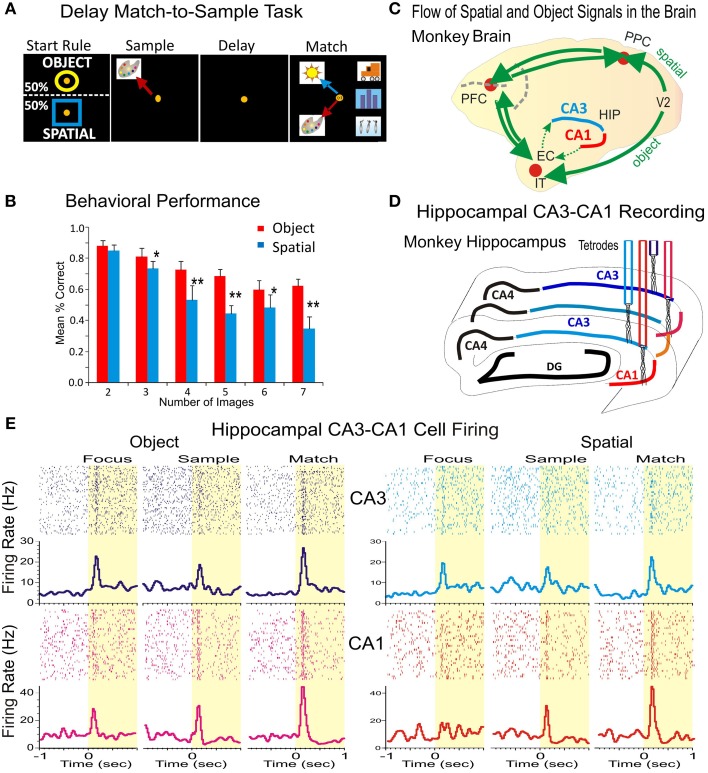
**Simultaneous recording of hippocampal CA1-CA3 neurons during the delayed match to sample task with variable number of images. (A)** Rule-based delayed match to sample task with variable number of objects (images). Distinct features of the task include: (1) the rule-based initial focus stimuli, (2) spatial location of the sample target, and (3) image features on object trials to be categorized, such as flowers, buildings animals or humans, (4) delays (1–60 s), and (5) the number of images in the match phase that randomly varies between 2 and 7. **(B)** Behavioral performance in the DMS task on Spatial and Object trials plotted as a function of the number of Clip-Art images presented on the screen for all animals from which recordings were obtained (*n* = 4). **(C)** Diagram showing the flow of spatial and object signals across the brain indicating connection of hippocampal subfields CA1-CA3 with prefrontal and parietal cortical areas. EC, entorhinal cortex; HIP, hippocampus; IT, inferotemporal cortex; PPC, posterior parietal cortex; PFC, prefrontal cortex; V2, secondary visual cortical area. **(D)** Diagram showing the locations for simultaneous recording from hippocampal subfields CA3-CA1 with four tetrodes. DG, dentate gyrus. **(E)** Individual rasters and peri-event firing activity of two simultaneously recorded cells from CA3 (blue) and CA1 (red) during the DMS task. Both cells display differential firing in response to all task events during the spatial and object trials of the task. ^*^p < 0.01, ^**^p < 0.001; ANOVA.

Four nonhuman primates (NHPs), were trained (Hampson et al., 2004a, 2013) to move into 25 cm clipart images on a 1.0 m × 1.0 m video projection screen by positioning the hand on a counter mounted to the chair, within a two-dimensional coordinate system that was video tracked by a fluorescent marker attached to the back of hand. Right limb (arm) position was tracked via an illuminated UV-fluorescent reflector affixed to the back of the hand and digitized and displayed as a large yellow cursor on the projection screen. Each monkey was trained to perform a visual delayed-match-to-sample (DMS) task for juice reinforcement. Animals performed 150–200 trials per 60–90 min DMS test session (Hampson et al., 2004a). The DMS task (Figure 1A and Figure 1S) consisted of a Sample and a Match phase in which an image presented in Sample phase was responded to and then a delay period of 10–90 s duration (selected at random) with the screen blanked and only the cursor illuminated. At termination of the delay interval, 1–6 nonmatch or “distracter” images were displayed together with the Sample image constituting the Match phase of the task in which placement of the cursor into the same image as presented in the Sample phase produced an immediate juice reward via a sipper tube positioned near the animals' mouth. Trials were separated by a variable 3 or 10 s inter–trial interval (ITI).

All animals were trained to a stable baseline performance level of 80–90% correct over all trials in a session (Figure 1B), however, as shown previously performance accuracy varied directly with difficulty or “cognitive workload” determined directly by the number of distracter images presented in the Match phase of the task (Hampson et al., 2004a). Sets of clip art images were changed frequently to maintain the trial-unique features of each session of the task and to prevent discriminative learning of image sets.

Experiments were designed such that object and spatial trials were presented randomly. Training and re-training was continued on a daily basis. The training was performed in steps, beginning with object contingency (yellow ring) and one image presentation, then the number of images was increased to 2 images for several weeks until performance was above the 80% threshold, and have repeated the same steps until the number of images was increased to 7 images. There was no interference between past and current tasks because the same task was employed continuously after the mechanics of the image dependent contingencies were performed adequately to allow presentation of all types of images in the same exact context throughout the time over which the results were presented. All animals performed to criteria and were monitored daily for changes related to task manipulations.

#### MIMO model for hippocampal neural activity during the DMS task

A multi-input/multi-output (MIMO) nonlinear dynamic model applied to spatiotemporal patterns of multiple recordings from primate hippocampal CA1 and CA3 neurons capable of extracting patterns of firing related to successful performance was applied to the same DMS memory task used here. The MIMO patterned stimulation was previously used to facilitate and recover performance when administered to the same locations as patterns of electrical pulses (Hampson et al., 2012a, 2013). This type of general Volterra kernel-based nonlinear model used in earlier studies (Berger et al., 2011; Hampson et al., 2012a,b,c, 2013; Opris and Ferrera, 2014) was employed to assess spatiotemporal nonlinear dynamics to predict CA1 output firing patterns via synaptic connectivity via input patterns of CA3 neural activity in primate hippocampus (Klausberger and Somogyi, 2008; Deguchi et al., 2011; Hampson et al., 2012d). The MIMO model was applied to recordings from the multiple tetrode probes described in Figure 1, and is structurally similar to the model shown to facilitate DMS performance when applied to NHP hippocampal and prefrontal cortical neurons in prior studies (Hampson et al., 2012a, 2013).

## Results

An intriguing question regarding the functional role of CA1 and CA3 microcircuits concerns how spatial and object information is actually processed by cells within these subfields that encode and retrieve memories (Hampson et al., 1999a, 2004a, 2013). To answer this question, four nonhuman primates (rhesus macaques) were trained to perform the delayed match to sample (DMS) task described above with the instruction to select in the Match Phase: (1) the retained features of Sample image, no matter what position was presented on the screen (object trials), or (2) the previously presented position of the image in the Sample phase irrespective of image features (spatial trials), as illustrated in Figure 1A and Figure 1S. The animals successfully executed arm-tracking movements to the appropriate visual targets for rewards in the Match phase of the DMS task that required selection of either the Sample image or position appropriately on object vs. spatial type trials. The DMS task incorporated key features of Sample target location, and the number of images (2–7) which could appear in any of eight locations on the screen in the Match phase after variable durations of the intervening delay period (10–90 s). These factors were reflected in the animal's behavioral performance levels during encoding and selection of object stimuli as shown in Figure 1B). There was no significant difference in behavioral performance determined with respect to the variance between individual animals. This showed that performance across animals was not significantly different from the mean values shown in Figure 1B.

Figure 1C shows how neural connections carrying task-related information in cortical areas and in hippocampal subfields CA3 and CA1. Figure 1D illustrates the recording paradigm for hippocampal subfields CA1-CA3. Neurons in CA3 and CA1 are interconnected in a distributed network between prefrontal, parietal and temporal cortices (Figure 1C) that processes spatial and object signals within the brain. Overall 677 neurons were recorded from the hippocampal CA1 (382 cells) and CA3 (295 cells) subfields in four animals. All of the recorded neurons that met the following criteria: (a) fired at rates higher than twice of baseline firing during the Sample, Match or both phase (*n* = 302∕382 cells in CA1, 79.06% and *n* = 240∕295 cells CA3, 81.35% of recorded cells); (b) showed significant spatial encoding during spatial trials in the Sample phase (*n* = 80∕302 cells in CA1, 26.49% and *n* = 55∕240 cells in CA3, 22.92% of recorded cells), (c) had significant firing activity during the Match phase on object trials (*n* = 165∕302 cells in CA1; 54.64% and *n* = 143∕240 cells in CA3, 59.58%) during image categorization and (*n* = 129∕302 cells, 42.72% in CA1 and *n* = 85∕240 cells, 35.42% of recorded cells in CA3) during numeric categorization. An illustration of such hippocampal cell firing in response to task contingency in CA3 and CA1 is shown in Figure 1E.

### Position encoding by hippocampal subfields CA1-CA3

**Figure 2:**
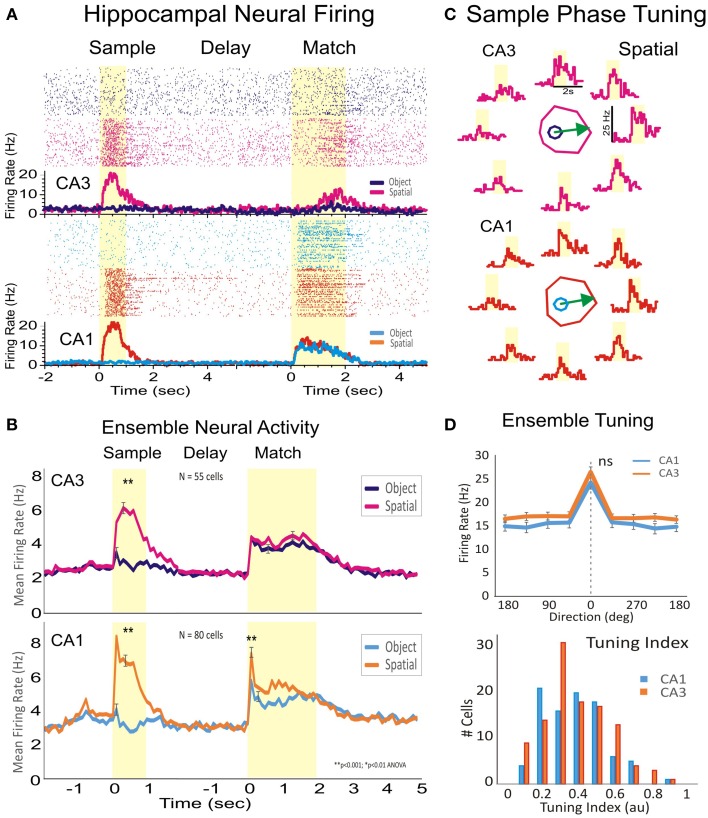
**Encoding of the spatial location of target images by hippocampal subfields CA1-CA3. (A)** Rasters and peri-event histogram depicting the firing of two hippocampal cells from CA1 and CA3 in response to the presentation of the sample, trial delay and presentation of the match phase. Both cells respond to sample target location when the spatial rule is in effect, but did not increase firing on object trials. **(B)** Firing for the subpopulation of hippocampal cells recorded from CA1 (*n* = 80) and from CA3 (*n* = 55) in four animals. While during sample presentation cells fired only during spatial trials and not to object trials, during the match phase the cells fired to both spatial and object cued stimuli. **(C)** Individual tuning plots for the two cells in **(A)** showing firing preference during sample presentation to the spatial location of the image on the right side of the screen. **(D)** Population tuning and tuning index of cells in CA3 and CA1 firing to a given spatial position of the Sample image. Error bars represent SEMs. Asterisks: ^**^p < 0.001, ANOVA. “ns” represents a nonsignificant statistic.

Hippocampal cell firing during the Sample phase of the DMS task reflects the encoding of stimulus features (spatial position vs. object (image) type), required for accurate retrieval and selection of the remembered target in the Match phase (see Figure 1A). Figure 2A shows the raster and peri-event histograms of two neurons recorded simultaneously from CA1 and CA3 with differential firing on Spatial and Object trials. Both cells responded only to the spatial cue but not to the object features during sample target presentation. In the Match phase the two cells respond differentially to both spatial location and object features. To assess spatial preference of the two cells during position encoding (in the Sample phase) in Figure 2B and Figure 2S is shown their spatial tuning (firing to preferred location), and both cells fired higher when the target was located on the right side of the screen.

As stated previously, subpopulations of neurons recorded in CA1 (*n* = 80 cells) and CA3 (n = 55 cells) exhibited significant peaks in mean firing (CA3: *z* = 22.58, *p* < 0.001 vs. CA1: *z* = 27.12, *p* < 0.001) during the Sample phase, but only on Spatial trials (Figure 2C). However, these same cells fired to both Spatial (CA3: *z* = 10.95, *p* < 0.001 vs. CA1: *z* = 19.42, *p* < 0.001) and Object (image) features (CA3: *z* = 9.28, *p* < 0.001 vs. CA1: *z* = 10.59, *p* < 0.001) during responding in the Match phase of the same trials. Thus, cells that fired during Sample presentation exclusively to encode spatial position, fired to both Spatial and Object stimuli during the Match phase. Figure 2D shows a similar overall spatial preference in the hippocampal subfields CA3 and CA1 (*p* >0.1, Rayleigh), together with the overall distribution of cells.

### Image feature and object trial categorization by hippocampal CA1-CA3 subfields

**Figure 3:**
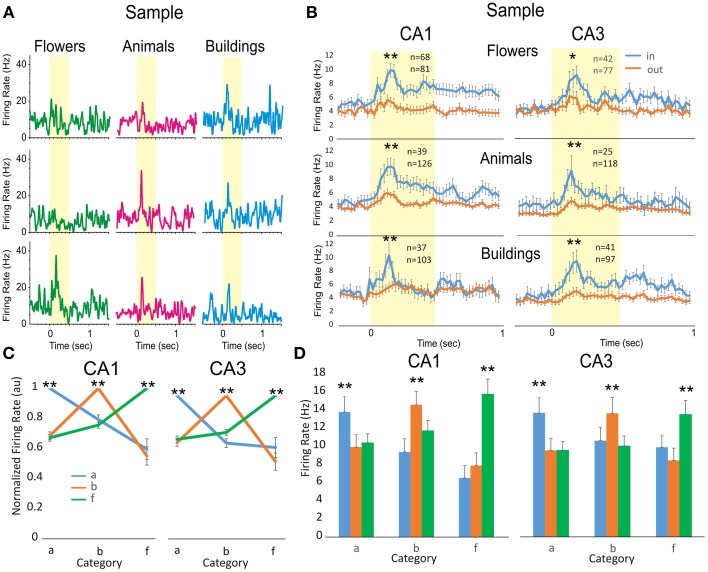
**Comparison of hippocampal firing to object image categorization. (A)** Three individual cells from hippocampal subfields CA3 and CA1 illustrate preferred category firing for Sample images that display either flowers (green), animals (pink), or buildings (blue) as one of the image features. **(B**). Population mean firing from all cells in hippocampal subfields CA3 (right) and CA1 (left) in response to images that have features in preferred (in) vs. nonpreferred (out) categories. The number of cells with significant response to the same (animals, buildings and flowers) categories from CA3 is (animals: *n* = 39^*^/126, buildings: 37^*^/103, and flowers: 68^*^/81) and from CA1 is (animals: *n* = 25^*^/118, buildings: 41^*^/97; flowers: 42^*^/77). In each group are counted the cells with significant firing in the subgroup of cells satisfying inclusion criteria in the category. **(C)** Normalized category preferred tuning of the population of cells in CA3 (*n* = 23, 33, 54) and CA1 (*n* = 30, 54, 53). **(D)** Population histograms showing mean firing peaks in each category, as compared to other categories, for the same population of cells in **Figure 4C**. Error bars represent SEMs. Asterisks: ^*^p < 0.01, ^**^p < 0.001; ANOVA.

Cells in the hippocampus of NHPs are known to categorize objects and/or screen images according to the features and number of items seen (Hampson et al., 2004a, 2013; Kourtzi and Connor, 2011). Figure 3 shows the categorization of features that appear in randomly selected screen clip-art images used in the DMS task such as: flowers (green), animals (pink) and buildings (blue). The three hippocampal cells shown in Figure 3A respond with higher firing rates to only one image category and with lower firing when the other two illustrated images occurred in the Sample phase. This trend is consistent at the population level in which a subset of cells from both hippocampal subfields (Figure 3B) identified previously as having specificity for Sample phase firing (see above), also responded significantly (CA1, *n* = 165 cells; CA3, *n* = 143 cells; *p* < 0.001, ANOVA) to only one of three assessed image categories (flowers, animals or buildings) presented on different trials. Category specific activity for this subset of cells is shown in Figures 3C,D for normalized and peak firing rates, respectively.

### Categorization of numeric features by hippocampal subfields CA1-CA3

The appearance of images on the screen in the Match phase varied from trial to trial which affected behavioral performance in a linear manner according to the number of images (2–7) from which the Sample (object or spatial position), had to be selected (Figure 1A). Behavioral performance was dependent on the number of “distracter” images presented (Figure 1B) which was also consistent with the increase in Match Response latency (reaction time + movement time) as the number of distracter images increased (Figure 1S). Hippocampal CA1&CA3 neural activity was also analyzed in relation to categorization as a function of number of images in the Match phase. The ratio of neurons recorded in each hippocampal subfield with enough trials to assess categorized Match phase firing was 129/302 in CA1 and 85/240 in CA3, and these combined showed activity that varied significantly [*F*_(1, 2139)_ = 12.85, *P* < 0.001, ANOVA] as a function of the number of images in the Match phase screen display on a given trial.

**Figure 4:**
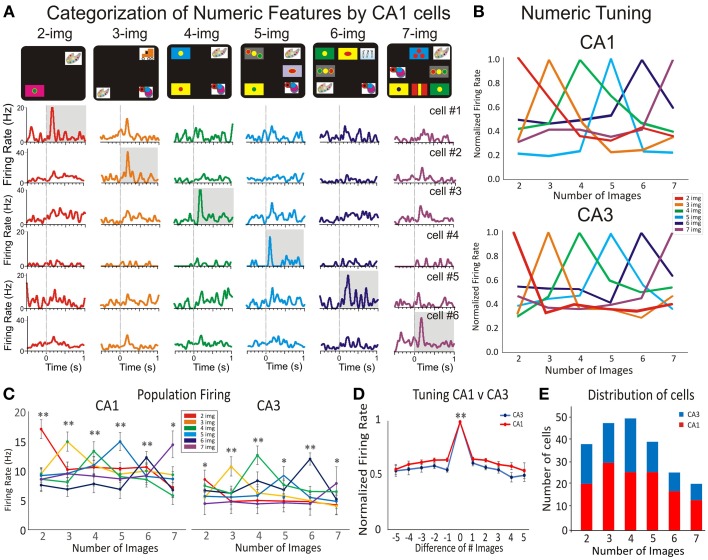
**Hippocampal subfields CA1-CA3 firing in response to numeric categorization**. Detection of numerosity features by numeric category selective hippocampal neurons. **(A)** Peri-event histogram arrays for six cells from CA1 that illustrate the selective numeric categorization of screen images. Thus, each row shows the firing response of one cell to the presentation of 2–7, images (in the Match phase) with the highest firing rate shaded in gray. Numeric categorization is illustrated by a color code so that red, orange, green, blue, dark blue and violet correspond to respectively 2, 3, 4, 5, 6, and 7 images on the screen. **(B)** The normalized activity for the cells in CA1 **(A)** and CA3 is shown for their selective category preference to the number of images. Note that both tuning plots to number categories (to CA1 and to CA3 in Figure 3S) show a distributed code. **(C)** The average firing activity across all selective cells in CA1 and CA3 with preferred numeric categories. Neurons in both subfields show significant firing preference for a given number of images. **(D)** Normalized average numeric tuning function across all preferred numeric categories and selective CA1 and CA3 neurons for the Match phase of the task. **(E)** Distributions of CA1 (red) and CA3 (blue) neurons with preferred numeric features in recorded during the Match phase of the DMS task. Error bars represent SEMs. Asterisks: ^*^p < 0.01, ^**^p < 0.001; ANOVA.

Figure 4A shows peri-event histograms of individual cells recorded in CA1, in which each row represents a distinct cell and each column represents the firing of different cells, when presented the same number of images, from 2 to 7). For each of these cells (CA1, *n* = 6) and (CA3, *n* = 6) the firing pattern was compared across the number of images in the Match phase (Figure 3S). Each cell shows peak activity for a particular number of images and a systematic drop-off of activity as the number varied from that preferred value (Hauser et al., 1996; Nieder, 2005). Figure 4B is shows “numeric tuning” of the preferred normalized activity (Piazza et al., 2004) of the same neurons from CA1 (top) and CA3 (bottom). To rule out the fact that firing could represent a particular screen shape or configuration other than the number of images, Figure 4S shows 2 hippocampal cells that fire preferentially to four images but have a different spatial tuning on the Match phase screen.

To evaluate this dependence across the population, the activity of each “number” category of selective neurons was plotted as a function of the number of images for both CA1 and CA3 (Figure 4C). Significant increases in firing activity (related to normalized baseline firing) of cells in CA1 and CA3 were obtained only on trials with the preferred number of images on the screen in the Match phase (CA1: *p* < 0.001, *n* = 129; CA3: *p* < 0.001, *n* = 85 cells; ANOVA) compared with trials with other image numbers in the Match phase.

Population neural filter functions (Nieder et al., 2002) were calculated by averaging the normalized activity for all neurons that preferred a given number of images. In Figure 4D we plotted activity as a function of distance from its preferred quantity (Nieder and Miller, 2004). On average, activity dropped off gradually with numerical distance (*p* < 0.001, Wilcoxon signed ranks test) for the Match phase CA1 (red) and CA3 (blue) intervals. Figure 4E shows that both hippocampal segments CA3 and CA1 show a steady distribution of preferred number categories in the match presentation phase.

### MIMO stimulation during sample phase in hippocampal ensemble CA1

**Figure 5:**
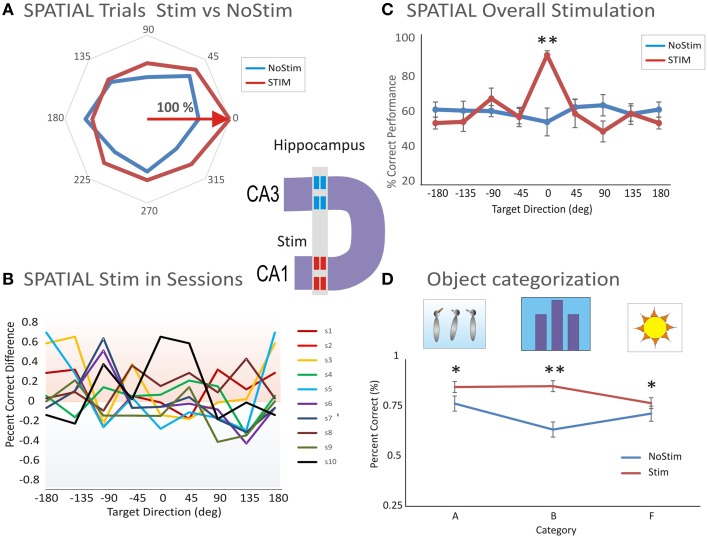
**Microstimulation induced facilitation on preferred target location encoding and categorization. (A)** Facilitated spatial tuning by delivery of MIMO stimulation. The facilitative effect of MIMO stimulation (Stim) vs. control (no-stim) trials shows both tuning and enhancement. **(B)** Performance difference between stimulation and control conditions across 10 sessions (s1–s10) illustrating the general trend of facilitated spatial tuning by MIMO Stim. **(C)** Comparison of the facilitation effect between MIMO stimulation and control conditions for Spatial tuning. Correct performance induced by MIMO stimulation (Stim) is compared with control (no-stim) conditions for cells that respond with spatial preferences (*n* = 20 sessions). **(D)** Comparison of the facilitation effect on categorization between MIMO stim (during the sample phase) and control (Nostim categorization). Altogether, the facilitated categorization and performance levels on MIMO Stim trials was significantly higher than the levels on no-stim trials (Facilitated vs. Control: *p* < 0.001; ANOVA). Error bars represent SEMs. Asterisks: ^*^p < 0.01, ^**^p < 0.001; ANOVA.

To further test whether hippocampal firing encoded the position of the sample target, or the numerical representation of the match phase screen, we applied the patterned stimulation previously shown to facilitate performance in the DMS task (Hampson et al., 2013). The application of the multi-input multi-output (MIMO) nonlinear model allowed extraction of a configuration of electrical (bipolar) stimulation pulses (20 uA amplitude and 1 ms duration) delivered to the same CA1 subfield from which task-related firing was obtained (Figure 1D and Figure 5S). This is shown as a functional diagram in which neural firing in hippocampal subfield CA3 was recorded with a multi-electrode array (Hampson et al., 2004b; Santos et al., 2012) and fed into a nonlinear multi-input–multi-output (MIMO) math model. Alter processing input signals from CA3, a pattern of electrical pulses (from a multi-channel stimulator that mimicked the output signals of correlated firing of CA1 cells) was simultaneously delivered to CA1 electrode locations, in the Sample phase on correct trials (Hampson et al., 2013). The diagram of the MIMO model driving a multichannel stimulator with output to CA1 electrode locations is illustrated in Figure 5S. Figure 5A shows an example of MIMO stimulation with induced behavioral tuning to a preferred spatial location (0°) compared to the No-stim trials. Figure 5B shows the performance difference in 10 sessions illustrating the general trend of facilitated spatial tuning (Figure 5B).

The effectiveness of MIMO stimulation delivered to the CA1 subfield is shown in Figure 5C where the preference effect on stimulated (Stim) vs. No-stim trials is compared for spatial codes in all (*n* = 20) stimulation sessions. The significant difference in mean % correct performance for MIMO Stim vs. NoStim trials is based on the underlying spatial preference [*F*_(1, 239)_ = 11.56, *p* < 0.001, ANOVA; Figure 5C].

Another effect of MIMO stimulation was to facilitate performance during categorization of object images during the Sample phase compared to similar categorizations on Nonstim trials. Altogether, the preference and correct performance levels on MIMO Stim trials were significantly higher than levels on no-stim trials [Facilitated vs. Control: *F*_(2, 238)_ = 12.78, *p* < 0.001, ANOVA; Figure 5D]. Thus, MIMO stimulation enhanced spatial location encoding and categorization ability of cells in the CA1 hippocampal subfield.

## Discussion

The primate hippocampus is widely regarded as the primary memory structure in mammalian brain with critical roles in encoding, representation, categorization and retrieval of cognitive information (McEchron and Disterhoft, 1999; Malkova and Mishkin, 2003; Squire et al., 2004; Rolls et al., 2005; Smith and Mizumori, 2006; Pastalkova et al., 2008; Hartley et al., 2013). It has been presumed that neural mechanisms underlying these cognitive functions use the input-output transformational properties of hippocampal laminar microcircuits in the subfields CA3 and CA1 (Sybirska et al., 2000; Förster et al., 2006). Consistent with this view, three crucial aspects dealing with encoding of (1) spatial location, (2) categorization of numeric features, and (3) distributed processing, in primate hippocampus, are demonstrated for the first time in the above descriptions.

### Encoding of spatial location

These findings provide new evidence about how spatial information is processed in the CA1 and CA3 subfields of primate hippocampus. Two groups of cells, in subfields CA3 and CA1 exhibited preferences for spatial location following the presentation of the Sample target (Figure 2), but only on Spatial and not on the Object trials. Spatial preference was further supported by MIMO model stimulation which increased correct performance on the same spatially preferred trials (Figures 5A,C) following delivery during Sample encoding on the same trials. Therefore, the facilitation effect of MIMO stimulation of hippocampal subfields in CA1 supports the role of these microcircuits in encoding spatial positions of targets in the task.

Hippocampal microcircuit cells in CA3 and CA1 undergo a marked change in firing during memory encoding of trial specific information that occurs immediately after trial rules switch from Spatial to Object. Once the rule code for the trial is known via response to the focus image, cells in the hippocampal microcircuits integrate and select signals corresponding to certain Sample image categories such as animals, buildings, flowers (as shown in Figure 3), or number of images (Figure 4). These findings provide insight into the neural basis of numeric categorization and processing in primate hippocampus.

### Categorization of numeric features and limitations

A second insight into the function of hippocampal microcircuits deals with the categorization of numeric features. One key aspect of numerical competence is the encoding and detection of multiple items (i.e., categorization). Both humans and animals have numeric competence (Nieder and Miller, 2004) and hippocampal cells have been shown to encode specific details of information within particular categories (Hampson et al., 1999a, 2004a, 2013) (hence categorization) within the match phase, based on the number of images (Objects), as shown here, confirms this function. This finding of numeric categorization in the primate hippocampus provides significant insight as to how it numerical selectivity in primate hippocampus is accomplished. As shown in Figures 3, 4 cells display higher firing for one of the screens containing a certain number of images and a systematic decrease in activity, as the number varied from the preferred value. This “numeric tuning” provides a signature of the preferred firing of the selective neurons forming “number” categories for all the trials (2–7 images) in the task. When compared across numeric categories, neural activity was proportionately attenuated as the numeric separation from the preferred value increased (Figure 4C). Moreover, facilitative MIMO model stimulation delivered during the Sample (encoding) phase of the task, was likely to have promoted categorization by CA1 neurons and that is what improved both spatial encoding and performance in the Match phase of the task. Although this study did not examine “numerosity” *per se*, the firing of hippocampal cells in Figure 5, exhibited statistically significant values for encoding and categorizing numeric features of image presentations (Object). This result is somewhat unique, especially because cognitive variables such as decision making, motor planning and reward expectation have been demonstrated by these same cells (Hampson et al., 2004a, 2013; Pennartz et al., 2011; Chen et al., 2013; McKenzie et al., 2014).

### Distributed encoding of spatial and object categories

A third important detail of categorization revealed by these findings is the distributed encoding of spatial and object categories in hippocampal CA1-CA3 neural ensembles (Eichenbaum and Fortin, 2003; Hafting et al., 2005; Naya and Suzuki, 2011). These results indicate that hippocampal neurons participate in high level, abstract visual representations by categorizing the object features and/or the number of images on a screen that distracts object selection. Contrary to the expectation drawn from single neuron studies in prefrontal and parietal cortices (Nieder et al., 2002; Nieder and Miller, 2004; Piazza et al., 2004; Nieder, 2005), some hippocampal neurons were tuned for the “number of items on a visual display” providing evidence that pyramidal neurons in both CA1 and CA3 are part of a distributed neural network primed to categorize visual features (Goldman-Rakic et al., 1984; Moser and Moser, 1998; Sybirska et al., 2000). These results support the idea of a distributed neuronal code for spatial and object categories in the primate brain including prefrontal, parietal, temporal cortices and hippocampus (Goldman-Rakic et al., 1984; Freedman and Assad, 2009; Carr and Frank, 2012; Wang et al., 2014). Such a distributed network for Spatial and Object categories with key nodes in prefrontal, parietal and temporal (hippocampus) cortices, integrates composite features of Objects in order to convey supra-modal invariance to sensory changes.

Spatial representation and its cellular basis in primate hippocampus has been previously described as “view cells” and object place cells by Rolls (1999). Such hippocampal “spatial view” neurons respond significantly different for different spatial views (centered in different frames) and encode information in their firing rates about the spatial view, but do not respond differently with respect to place, head direction or eye position (Rolls et al., 1997; Robertson et al., 1998). Rolls et al. (2005) demonstrated that an episodic memory system with separate and combined neuronal representations of objects and where they are seen “out there” in the environment, is present in the primate hippocampus. The results shown here indicate that spatial representation can be dissociated from object features.

Finally, it is important to recognize that the transformational properties of hippocampal microcircuitry, together with the patterned microstimulation, applied during the sample image presentation facilitated selection of either sample image spatial locations or types of images. MIMO model stimulation which mimicked the endogenous specific pattern, markedly improved categorization performance (Figure 5D) is supporting the practical importance of this approach for cognitive enhancement and rehabilitation, such as in memory neuroprosthetics (Opris, 2013).

## Conclusion

These unique results show for the first time that CA3 and CA1 hippocampal neurons in primate brain encode spatial information during the Sample phase and categorize numeric features during the Match phase of DMS task (Freedman et al., 2001; Porrino et al., 2005; Deadwyler et al., 2007; Hampson et al., 2013). These crucial aspects dealing with encoding of spatial location, and categorization of numeric features appear to be systematically distributed within primate hippocampal CA1-CA3 subfields. Furthermore, applying task and performance specific MIMO model microstimulation was capable of enhancing encoding of spatial and object features. These findings support the existence of distributed neuronal codes for spatial and object cognitive information in primate hippocampus (Freedman et al., 2001; Rolls et al., 2005). Moreover, these results support the importance of this approach for cognitive enhancement and rehabilitation (Opris, 2013), especially relevant for memory neuroprosthetics.

## Author contributions

IO designed the research, supervised the outcome of MEA recordings, performed data analysis and wrote the manuscript. LS performed tetrode recordings; GG provided MEA for simultaneous recording in hippocampal CA1–CA3 technical assistance; DS and TB provided the MIMO model and the programming and microelectronics needed for microstimulation. RH designed the experiments, provided technical expertise and supervised the project. SD designed the experiments, provided expertise with all aspects of the hippocampal system.

### Conflict of interest statement

The authors declare that the research was conducted in the absence of any commercial or financial relationships that could be construed as a potential conflict of interest.

## References

[B1] Agarwal G. Stevenson I. H. Berényi A. Mizuseki K. Buzsáki G. Sommer F. T. (2014). Spatially distributed local fields in the hippocampus encode rat position. Science 344, 626–630. 10.1126/science.1250444 24812401 PMC4909490

[B2] Amaral D. G. Lavenex P. (2007). The Hippocampus Book. New York, NY: Oxford University Press.

[B3] Berger T. W. Hampson R. E. Song D. Goonawardena A. Marmarelis V. Z. Deadwyler S. A. (2011). A cortical neural prosthesis for restoring and enhancing memory. J. Neural. Eng. 8:046017. 10.1088/1741-2560/8/4/046017 21677369 PMC3141091

[B4] Brun V. H. Otnass M. K. Molden S. Steffenach H. A. Witter M. P. Moser M. B.. (2002). Place cells and place recognition maintained by direct entorhinal-hippocampal circuitry. Science 296, 2243–2246. 10.1126/science.1071089 12077421

[B5] Carr M. F. Frank L. M. (2012). A single microcircuit with multiple functions: state dependent information processing in the hippocampus. Curr. Opin. Neurobiol. 22, 704–708. 10.1016/j.conb.2012.03.007 22480878 PMC3438355

[B6] Chen G. King J. A. Burgess N. O'Keefe J. (2013). How vision and movement combine in the hippocampal place code. Proc. Natl. Acad. Sci. U.S.A. 110, 378–383. 10.1073/pnas.1215834110 23256159 PMC3538268

[B7] Deadwyler S. A. Porrino L. Siegel J. M. Hampson R. E. (2007). Systemic and nasal delivery of orexin-A (Hypocretin-1) reduces the effects of sleep deprivation on cognitive performance in nonhuman primates. J. Neurosci. 27, 14239–14247. 10.1523/JNEUROSCI.3878-07.2007 18160631 PMC6673447

[B8] Deguchi Y. Donato F. Galimberti I. Cabuy E. Caroni P. (2011). Temporally matched subpopulations of selectively interconnected principal neurons in the hippocampus. Nat. Neurosci. 14, 495–504. 10.1038/nn.2768 21358645

[B9] Eichenbaum H. Fortin N. (2003). Episodic memory and the hippocampus: it's about time Curr. Dir. Psychol. Sci. 12, 53–57. 10.1111/1467-8721.01225

[B10] Förster E. Zhao S. Frotscher M. (2006). Laminating the hippocampus. Nat. Rev. Neurosci. 7, 259–268. 10.1038/nrn1882 16543914

[B11] Freedman D. J. Assad J. A. (2009). Distinct encoding of spatial and nonspatial information in parietal cortex. J. Neurosci. 29, 5671–5680. 10.1523/JNEUROSCI.2878-08.2009 19403833 PMC2898938

[B12] Freedman D. J. Riesenhuber M. Poggio T. Miller E. K. (2001). Categorical representation of visual stimuli in the primate prefrontal cortex. Science 291:312. 10.1126/science.291.5502.312 11209083

[B13] Goldman-Rakic P. S. Selemon L. D. Schwartz M. L. (1984). Dual pathways connecting the dorsolateral prefrontal cortex with the hippocampal formation and parahippocampal cortex in the rhesus monkey. Neuroscience 12, 719–743. 10.1016/0306-4522(84)90166-0 6472617

[B14] Hafting T. Fyhn M. Molden S. Moser M. B. Moser E. I. (2005). Microstructure of a spatial map in the entorhinal cortex. Nature 436, 801–806. 10.1038/nature03721 15965463

[B15] Hampson R. E. Coates T. D. Jr. Gerhardt G. A. Deadwyler S. A. (2004b). Ceramic-based micro-electrode neuronal recordings in the rat and monkey. Proc. Annu. Int. Conf. IEEE Eng. Med. Biol. Soc. 25, 3700–3703. 10.1109/IEMBS.2003.1280962

[B16] Hampson R. E. Gerhardt G. A. Marmarelis V. Z. Song D. Opris I. Santos L.. (2012a). Facilitation and restoration of cognitive function in primate prefrontal cortex by a neuroprosthesis that utilizes minicolumn-specific neural firing. J. Neural. Eng. 9:056012. 10.1088/1741-2560/9/5/056012 22976769 PMC3505670

[B17] Hampson R. E. Jarrard L. E. Deadwyler S. A. (1999b). Effects of ibotenate hippocampal and extrahippocampal destruction on delayed-match and -nonmatch-to-sample behavior in rats. J. Neurosci. 19, 1492–1507. 9952425 10.1523/JNEUROSCI.19-04-01492.1999 PMC6786034

[B18] Hampson R. E. Opris I. Song D. Gerhardt G. A. Shin D. Marmarelis V. Z. (2012b). Neural representation of cognitive processing in the prefrontal cortex of nonhuman primates, in Conference Proceedings of the IEEE Engineering in Medicine and Biology Society. 10.1109/EMBC.2012.6346485 23366446

[B19] Hampson R. E. Pons T. P. Stanford T. R. Deadwyler S. A. (2004a). Categorization in the monkey hippocampus: a possible mechanism for encoding information into memory. Proc. Natl. Acad. Sci. U.S.A. 101, 3184–3189. 10.1073/pnas.0400162101 14978264 PMC365764

[B20] Hampson R. E. Simeral J. D. Deadwyler S. A. (1999a). Distribution of spatial and nonspatial information in dorsal hippocampus. Nature 402, 610–614. 10.1038/45154 10604466

[B21] Hampson R. E. Song D. Chan R. H. Sweatt A. J. Riley M. R. Gerhardt G. A.. (2012c). A nonlinear model for hippocampal cognitive prosthesis: memory facilitation by hippocampal ensemble stimulation. IEEE Trans. Neural Syst. Rehabil. Eng. 20, 184–197. 10.1109/TNSRE.2012.2189163 22438334 PMC3397311

[B22] Hampson R. E. Song D. Chan R. H. Sweatt A. J. Riley M. R. Goonawardena A. V.. (2012d). Closing the loop for memory prosthesis: detecting the role of hippocampal neural ensembles using nonlinear models. IEEE Trans. Neural. Syst. Rehabil. Eng. 20, 510–525. 10.1109/TNSRE.2012.2190942 22498704 PMC3395725

[B23] Hampson R. E. Song D, Opris, I, Santos, L. M. Shin D. C. Gerhardt G. A.. (2013). Facilitation of memory encoding in primate hippocampus by a neuroprosthesis that promotes task specific neural firing. J. Neural. Eng. 10:066013. 10.1088/1741-2560/10/6/066013 24216292 PMC3919468

[B24] Hartley T. Lever C. Burgess N. O'Keefe J. (2013). Space in the brain: how the hippocampal formation supports spatial cognition. Philos. Trans. R. Soc. Lond. B Biol. Sci. 369, 20120510. 10.1098/rstb.2012.0510 24366125 PMC3866435

[B25] Hauser M. D. MacNeilage P. Ware M. (1996). Numerical representations in primates. Proc. Natl. Acad. Sci. U.S.A. 93, 1514–1517. 10.1073/pnas.93.4.1514 8643663 PMC39971

[B26] Hirabayashi T. Miyashita Y. (2014). Computational principles of microcircuits for visual object processing in the macaque temporal cortex. Trends Neurosci. 37, 178–187. 10.1016/j.tins.2014.01.002 24491832

[B27] Klausberger T. Somogyi P. (2008). Neuronal diversity and temporal dynamics: the unity of hippocampal circuit operations. Science 321, 53–57. 10.1126/science.1149381 18599766 PMC4487503

[B28] Kourtzi Z. Connor C. E. (2011). Neural representations for object perception: structure, category, and adaptive coding. Annu. Rev. Neurosci. 34, 45–67. 10.1146/annurev-neuro-060909-153218 21438683

[B29] Lavenex P. Lavenex P. B. Amaral D. G. (2006). Spatial relational learning persists following neonatal hippocampal lesions in macaque monkeys. Nat. Neurosci. 10, 234–239. 10.1038/nn1820 17195843

[B30] Malkova L. Mishkin M. (2003). One-trial memory for object–place associations after separate lesions of hippocampus and osterior parahippocampal region in the monkey. J. Neurosci. 23, 1956–1965. 12629201 10.1523/JNEUROSCI.23-05-01956.2003 PMC6741967

[B31] McEchron M. D. Disterhoft J. F. (1999). Hippocampal encoding of non-spatial trace conditioning. Hippocampus 9, 385–396. 10495020 10.1002/(SICI)1098-1063(1999)9:4<385::AID-HIPO5>3.0.CO;2-K

[B32] McKenzie S. Frank A. J. Kinsky N. R. Porter B. Rivière P. D. Eichenbaum H. (2014). Hippocampal representation of related and opposing memories develop within distinct, hierarchically organized neural schemas. Neuron 83, 202–215. 10.1016/j.neuron.2014.05.019 24910078 PMC4082468

[B33] Mishkin M. Suzuki W. A. Gadian D. G. Vargha-Khadem F. (1997). Hierarchical organization of cognitive memory. Philos. Trans. R. Soc. Lond. B Biol. Sci. 352, 1461–1467. 10.1098/rstb.1997.0132 9368934 PMC1692056

[B34] Moser M. Moser E. I. (1998). Distributed encoding and retrieval of spatial memory in the hippocampus. J. Neurosci. 18, 7535–7542. 9736671 10.1523/JNEUROSCI.18-18-07535.1998 PMC6793256

[B35] Munoz M. Chadwick M. Perez-Hernandez E. Vargha-Khadem F. Mishkin M. (2011). Novelty preference in patients with developmental amnesia. Hippocampus 21, 1268–1276. 10.1002/hipo.20836 20882542 PMC3021098

[B36] Murray E. A. Baxter M. G. Gaffan D. (1998). Monkeys with rhinal cortex damage or neurotoxic hippocampal lesions are impaired on spatial scene learning and object reversals. Behav. Neurosci. 112, 1291–1303. 10.1037/0735-7044.112.6.1291 9926813

[B37] Naya Y. Suzuki W. A. (2011). Integrating what and when across the primate medial temporal lobe. Science 333, 773–776. 10.1126/science.1206773 21817056

[B38] Nieder A. (2005). Counting on neurons: the neurobiology of numerical competence. Nat. Rev. Neurosci. 6, 177–190. 10.1038/nrn1626 15711599

[B39] Nieder A. Freedman D. J. Miller E. K. (2002). Representation of the quantity of visual items in the primate prefrontal cortex. Science 297, 1708–1711. 10.1126/science.1072493 12215649

[B40] Nieder A. Miller E. K. (2004). A parieto-frontal network for visual numerical information in the monkey. Proc. Natl. Acad. Sci. U.S.A. 101, 7457–7462. 10.1073/pnas.0402239101 15123797 PMC409940

[B41] O'Keefe J. Dostrovsky J. (1971). The hippocampus as a spatial map. Preliminary evidence from unit activity in the freely-moving rat. Brain Res. 34, 171–175. 10.1016/0006-8993(71)90358-1 5124915

[B42] Opris I. (2013). Inter-laminar microcircuits across the neocortex: repair and augmentation. Front. Syst. Neurosci. 7:80. 10.3389/fnsys.2013.00080 24312019 PMC3832795

[B43] Opris I. Ferrera V. P. (2014). Modifying cognition and behavior with electrical microstimulation: implications for cognitive prostheses. Neurosci. Biobehav. Rev. 47, 321–335. 10.1016/j.neubiorev.2014.09.003 25242103

[B44] Pagano S. Mazza V. (2013). Multiple object individuation during numerical Stroop. Psychophysiology 50, 292–296. 10.1111/psyp.12014 23317034

[B45] Pastalkova E. Itskov V. Amarasingham A. Buzsáki G. (2008). Internally generated cell assembly sequences in the rat hippocampus. Science 321, 1322–1327. 10.1126/science.1159775 18772431 PMC2570043

[B46] Pennartz C. M. Ito R. Verschure P. F. Battaglia F. P. Robbins T. W. (2011). The hippocampal-striatal axis in learning, prediction and goal-directed behavior. Trends Neurosci. 34, 548–559. 10.1016/j.tins.2011.08.001 21889806

[B47] Piazza M. Izard V. Pinel P. Le Bihan D. Dehaene S. (2004). Tuning curves for approximate numerosity in the human intraparietal sulcus. Neuron 44, 547–555. 10.1016/j.neuron.2004.10.014 15504333

[B48] Porrino L. J. Daunais J. B. Rogers G. A. Hampson R. E. Deadwyle S. A. (2005). Facilitation of task performance and removal of the effects of sleep deprivation by an ampakine (CX717) in nonhuman primates. PLoS Biol. 3:e299. 10.1371/journal.pbio.0030299 16104830 PMC1188239

[B49] Robertson R. G. Rolls E. T. Georges-François P. (1998). Spatial view cells in the primate hippocampus: effects of removal of view details. J. Neurophysiol. 79, 1145–1156. 9497397 10.1152/jn.1998.79.3.1145

[B50] Rolls E. T. (1999). Spatial view cells and the representation of place in the primate hippocampus. Hippocampus 9, 467–480. 10495028 10.1002/(SICI)1098-1063(1999)9:4<467::AID-HIPO13>3.0.CO;2-F

[B51] Rolls E. T. Treves A. Foster D. Perez-Vicente C. (1997). Simulation studies of the CA3 hippocampal subfield modelled as an attractor neural network. Neural Netw. 10, 1559–1569. 10.1016/S0893-6080(97)00092-0

[B52] Rolls E. T. Xiang J.-Z. Franco L. (2005). Object, space, and object-space representations in the primate hippocampus. J. Neurophysiol. 94, 833–844. 10.1152/jn.01063.2004 15788523

[B53] Sagiv N. Ward J. (2006). Crossmodal interactions: lessons from synesthesia. Prog. Brain Res. 155, 259–271. 10.1016/S0079-6123(06)55015-0 17027393

[B54] Santos L. M. Opris I. Fuqua J. Hampson R. E. Deadwyler S. A. (2012). A novel tetrode microdevice for simultaneous single-unit recording and microstimulation of several brain regions in primates. J. Neurosci. Methods 205, 368–374. 10.1016/j.jneumeth.2012.01.006 22326226 PMC3342772

[B55] Scoville W. B. Milner B. (1957). Loss of recent memory after bilateral hippocampal lesions. J. Neurol. Neurosurg. Pysciatry 20, 11–12. 10.1136/jnnp.20.1.11 13406589 PMC497229

[B56] Smith C. N. Urgolites Z. J. Hopkins R. O. Squire L. R. (2014). Comparison of explicit and incidental learning strategies in memory-impaired patients. Proc. Natl. Acad. Sci. U.S.A. 111, 475–479. 10.1073/pnas.1322263111 24367093 PMC3890873

[B57] Smith D. M. Mizumori S. J. (2006). Learning-related development of context-specific neuronal responses to places and events: the hippocampal role in context processing. J. Neurosci. 26, 3154–3163. 10.1523/JNEUROSCI.3234-05.2006 16554466 PMC6674095

[B58] Snyder A. C. Shpaner M. Molholm S. Foxe J. J. (2012). Visual object processing as a function of stimulus energy, retinal eccentricity and Gestalt configuration: a high-density electrical mapping study. Neuroscience 27, 1–11. 10.1016/j.neuroscience.2012.03.035 22521825

[B59] Squire L. R. Clark R. E. Bailey P. J. (2004). The Cognitive Neuroscience, 3rd Edn. Cambridge, MA: MIT Press.

[B60] Sybirska E. Davachi L. Goldman-Rakic P. S. (2000). Prominence of direct entorhinal–CA1 pathway activation in sensorimotor and cognitive tasks revealed by 2-DG functional mapping in nonhuman primate. J. Neurosci. 20, 5827–5834. 10908624 10.1523/JNEUROSCI.20-15-05827.2000 PMC6772564

[B61] Wang J. X. Rogers L. M. Gross E. Z. Ryals A. J. Dokucu M. E. Brandstatt K. L.. (2014). Targeted enhancement of cortical-hippocampal brain networks and associative memory. Science 345, 1054. 10.1126/science.1252900 25170153 PMC4307924

[B62] Zola S. M. Squire L. R. (2001). Relationship between magnitude of damage to the hippocampus and impaired recognition memory in monkeys. Hippocampus 11, 92–98. 10.1002/hipo.1027 11345130

