# Supplementary material for: Distributed encoding of spatial and object categories in primate hippocampal microcircuits
Source: Front Neurosci. 2015 Oct 6;9:317. doi: 10.3389/fnins.2015.00317 (PMC4594006; doi:10.3389/fnins.2015.00317)
Supplement: Supplementary file 1 [file DataSheet1.PDF]

SUPPLEMENTARY INFORMATION

Distributed Encoding of Spatial and Object Categories in Primate Hippocampal Microcircuits

Ioan Opris[1], Lucas M. Santos[1], Greg A. Gerhardt[2], Dong Song[3], Theodore W Berger[3], Robert E. Hampson[1] and Sam A. Deadwyler[1]

ONLINE METHODS

All animal procedures were reviewed and approved by the Institutional Animal Care and Use Committee (ACUC) of Wake Forest Baptist Health University, in accordance with guidelines of the U.S. Department of Agriculture, American Association of Laboratory Animal Care, and National Institutes of Health.

Behavioral Task: Four nonhuman primates (NHPs), weighing 8.0–11.0 kg were trained to sit in primate chairs in front of a display screen[5,6,53,55] and move a cursor with their right arm and hand. Right limb (arm) position was tracked via an illuminated UV-fluorescent reflector affixed to the back of the hand and digitized and displayed as a large yellow cursor on the projection screen. Each monkey was trained to perform a visual delayed-match-to-sample (DMS) task for juice reinforcement and tested daily 5 times per week. Animals were seated in a primate chair 1.5m in front of an LCD-front-projection screen and performed 150–200 trials per 60–90min DMS test session[5,55-57]. They were trained to move a cursor into 25 cm clipart images on a 1.0m×1.0m video projection screen by positioning the hand on a counter mounted to the chair, within a two-dimensional coordinate system that was video tracked by a fluorescent marker attached to the back

of hand. The DMS task (Fig. 1A & 1S) consisted of a Sample and a Match phase in which an image presented in Sample phase was responded to and then a delay period of 10–90s duration (selected at random) with the screen blanked and only the cursor illuminated. At termination of the delay interval, 1–6 nonmatch or “distracter” images were displayed together with the Sample image constituting the Match phase of the task in which placement of the cursor into the same image as presented in the Sample phase produced an immediate juice reward via a sipper tube positioned near the animals’ mouth. Trials were separated by a variable 3 or 10s inter–trial interval (ITI). Each animal was trained at least one year and recorded from 2-3 times per week.

All displayed clip art images used in the DMS task were unique to a particular trial, and no image was presented on more than one trial within an entire session.. All animals were trained to a stable baseline performance level of 80–90% correct over all trials in a session (Fig 1B), however, as shown previously performance accuracy varied directly with difficulty or “cognitive workload” determined directly by the number of distracter images presented in the Match phase of the task[5,55-58]. Sets of clip art images were changed frequently to maintain the trial-unique features of each session of the task and to prevent discriminative learning of image sets.

Experiments were designed such that object and spatial trials were presented randomly. Training and re-training was continued on a daily basis. The training was performed in steps, beginning with object contingency (yellow ring) and one image presentation, then the number of images was increased to 2 images for several weeks until performance was above the 80% threshold, and have repeated the same steps until

the number of images was increased to 7 images. There was no interference between past and current tasks because the same task was employed continuously after the mechanics of the image dependent contingencies were performed adequately to allow presentation of all types of images in the same exact context throughout the time over which the results were presented. All animals performed to criteria and were monitored daily for changes related to task manipulations.

Surgery: Each animal (rhesus monkeys – macaca mulatta), was anesthetized with ketamine (10 mg/kg), then intubated and maintained with isoflurane (1-2 % in oxygen 6 l/min) then surgically prepared with cylinders for attachment of a microelectrode manipulator over the specified brain regions of interest. Recording cylinders (Crist Instruments) were placed over 20 mm diameter craniotomies for electrode access[5,55- 58] selected to overlie the stereotaxic coordinates of hippocampus (12 mm anterior to interaural line, 12 mm lateral to midline[59]. (Fig. 1D). Two titanium posts were secured to the skull for head restraint. Following surgery, animals were given 0.025 mg/kg buprenorphine for analgesia and penicillin to prevent infection. Cylinders are cleaned weekly with betadine during surgical recovery and daily recording sessions.

Recording Procedures: Recording and analysis software for 64 channel simultaneous recordings utilized the MAP Spike Sorter by Plexon, Inc (Dallas, TX). Customized tetrodes[22], were manufactured specifically for recording spatially distinct locations in the CA3 and CA1 cell fields in primate hippocampus[5] such that multi-cell ($n>12$) recordings could be obtained from each anatomically distinct location. The Schaefer collateral

projections from CA3 to CA1 are ubiquitous enough to insure that the locations recorded from in CA3 were likely connected synaptically to the locations recorded from in CA1 in each tetrode pair located in the same mediolateral plane or "chip" of hippocampus in two distinct anterior posterior locations as shown in Figure 1[22,55]. This tetrode arrangement insured that only cells in CA3 and CA1 were isolated and recorded, since the appearance of activity on each vertically inserted probe occurred at depths of insertion for CA1 that required prior traversal through cell layer CA3 placed in the same cross-sectional plane of hippocampus as shown in Figure 1D.

***Data Analysis*.** Task performance was determined for each animal (n=4) as % correct responses within trial groups sorted according to duration of delay and number of images presented in Match phase of the task (Figure 1B&C). Number of correct and incorrect trials were summed and percentages computed within sessions, across a minimum of three sessions[60]. Recordings of multiple CA3 and CA1 neuron firings on individual trials[5] during the Sample and Match phases of the DMS task were summed within 100 ms bins, and accumulated across trials within a session for display as perievent histograms (PEHs) of mean firing rate (i.e. spikes/sec) relative to the Sample or Match events (Figures 2&3). Cell types were identified as regular firing hippocampal cells in terms of baseline (nonevent) firing rate[5,57,61] and peaks on single trials in PEHs derived for intervals of ± 2.0s relative to the onset of the screen image display (0.0s) in the Sample and Match phases of the task. Significant firing peaks were identified by maximum firing rate ± 0.5s relative to the DMS event. Neurons were only included in the analysis if their firing rates were significantly elevated (Z-scores, ANOVA F test $p < 0.01$) relative to the pre-event

screen presentation baseline period (-2.0 to 0.0s). Finally, the correspondence of firing between cells in different layers was tested via comparison of trial-based histograms (TBHs) spanning more than one task event within a phase to construct templates related to how the hippocampus encoded trial specific information. PEHs demarcated firing differences for individual events and to provide the basis for nonlinear model analyses of firing during particular Sample and Match types of events within a given trial. Categorization was done according to the specific content of sample images that were responded to with increased firing across different images. Categories were formed by association of common features in multiple different images that grouped and confirmed by the experimenters in multiple subsequent presentation of the same images- essentially the images are inspected to see common features that are present in each image that is responded to and provokes increased cell firing. Categorization is based on these consistent similarities in performance and cell firing across thousands of trials with images that differ but have the same elements (i.e. buildings. flowers, animals, humans etc.

Tuning Plots. For each analyzed neuron, firing was aligned to presentation of the sample target position selected. Directionality was assigned according to 8 different "clock" directions corresponding to the location of the match image around the periphery of the screen, yielding 0°, 45°, 90°, 135°, 180°, 225°, 270°, 315° & 360° movement directions (from center of screen). Mean firing rate after sample presentation but immediately prior to sample response (i.e. 0.0-1.0 s) for each response position was calculated and represented in polar coordinates as a tuning vector[62,63]. The directional bias for a given cell was revealed by Match Response locations with the highest mean firing rate *and* by the direction of tuning vectors computed during the Match Phase (Figs 2 and 2S, polar plots). Similarly, tuning

vectors were plotted for percent correct performance under stimulation vs. no stimulation conditions. To quantify tuning polygonal contours we used overlap values between 0 (totally dissimilar) and 1 (identical, no statistical significance) and Rayleigh test for statistical significance[28-30,62].

MIMO Model for Hippocampal Neural Activity during the DMS Task Prior studies[60,64] have shown that a multi-input/multi-output (MIMO) nonlinear dynamic model applied to spatiotemporal patterns of multiple recordings from primate hippocampal CA1 and CA3 neurons capable of extracting patterns of firing related to successful performance was applied to the same DMS memory task used here. The MIMO patterned stimulation was previously used to facilitate and recover performance when administered to the same locations as patterns of electrical pulses[6,60]. This type of general Volterra kernel-based nonlinear model used in earlier studies[6,60,64-67] was employed to assess spatiotemporal nonlinear dynamics to predict CA1 output firing patterns via synaptic connectivity via input patterns of CA3 neural activity in primate hippocampus[68-70]. The MIMO model was applied to recordings from the multiple tetrode probes described in Figure 1, and is structurally similar to the model shown to facilitate DMS performance when applied to NHP hippocampal and prefrontal cortical neurons in prior studies[6,60].

Supplementary Figures

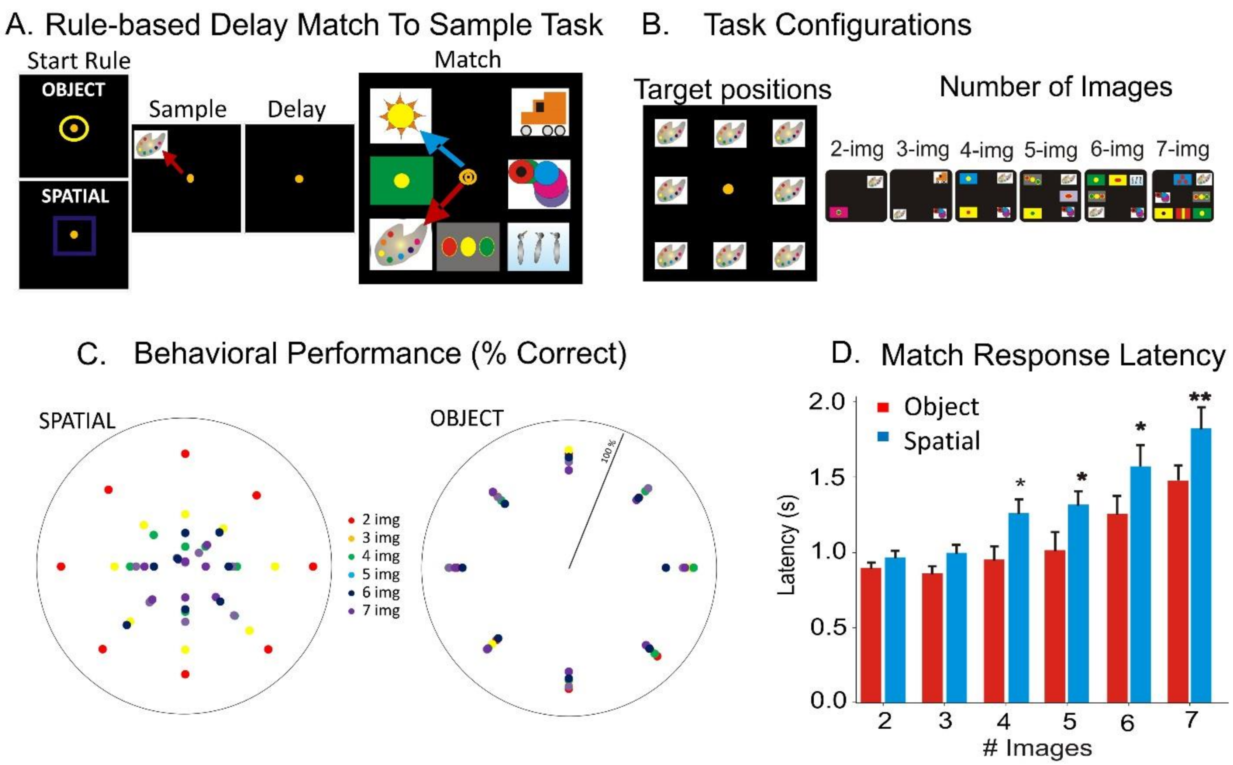

Figure 1S. **Behavioral Paradigm and Performance. Delayed-Match-to-Sample (DMS) task with variable number of objects. A**) Rule-based DMS task with variable number of objects (images). Start Rules show the focus images (Object circle and Spatial square) that are required to be activated to start each trial and they depict what "rule" is applied for successful responding in the Match phase relative to the Image presented in the Sample phase. A Spatial trial requires a Match Response (MR) in the same screen position as the Sample Response (SR), whereas an Object trial requires selection in the Match phase of the same image presented in the Sample phase regardless of where on the screen and in conjunction with the presence of 1-6 other distracter images. Relevant for this study are the locations of the sample targets and the number of images presented in the match phase that randomly varies between 2 and 7. **B**) DMS task configuration. Display of the eight spatial target locations and the screen with 2-7 number of images. **C**) Behavioral

performance in the DMS task during spatial (left) and object (right) trials plotted in polar coordinates as a function of the number of images/objects on the screen. **D)** Match response latency (indicated as time from the presentation of the Match screen to selection of the target) during spatial vs. object trials plotted as a function of number of images on the screen.

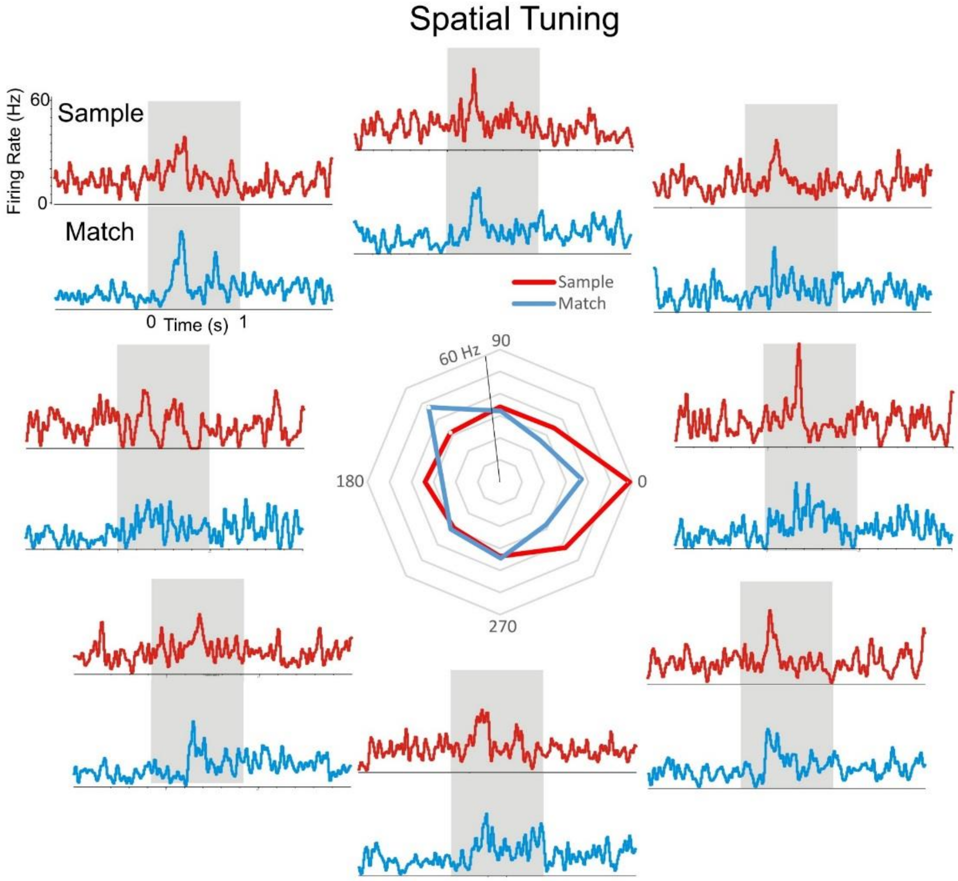

Figure 2S Spatial Tuning. Multigram with peri-event firing plotted across the eight different target image locations, with tuning plots in the center to compare spatial firing of a single hippocampal (CA1) cell for each target location during the sample and match phases of the DMS task to illustrate preferences and the distributed code.

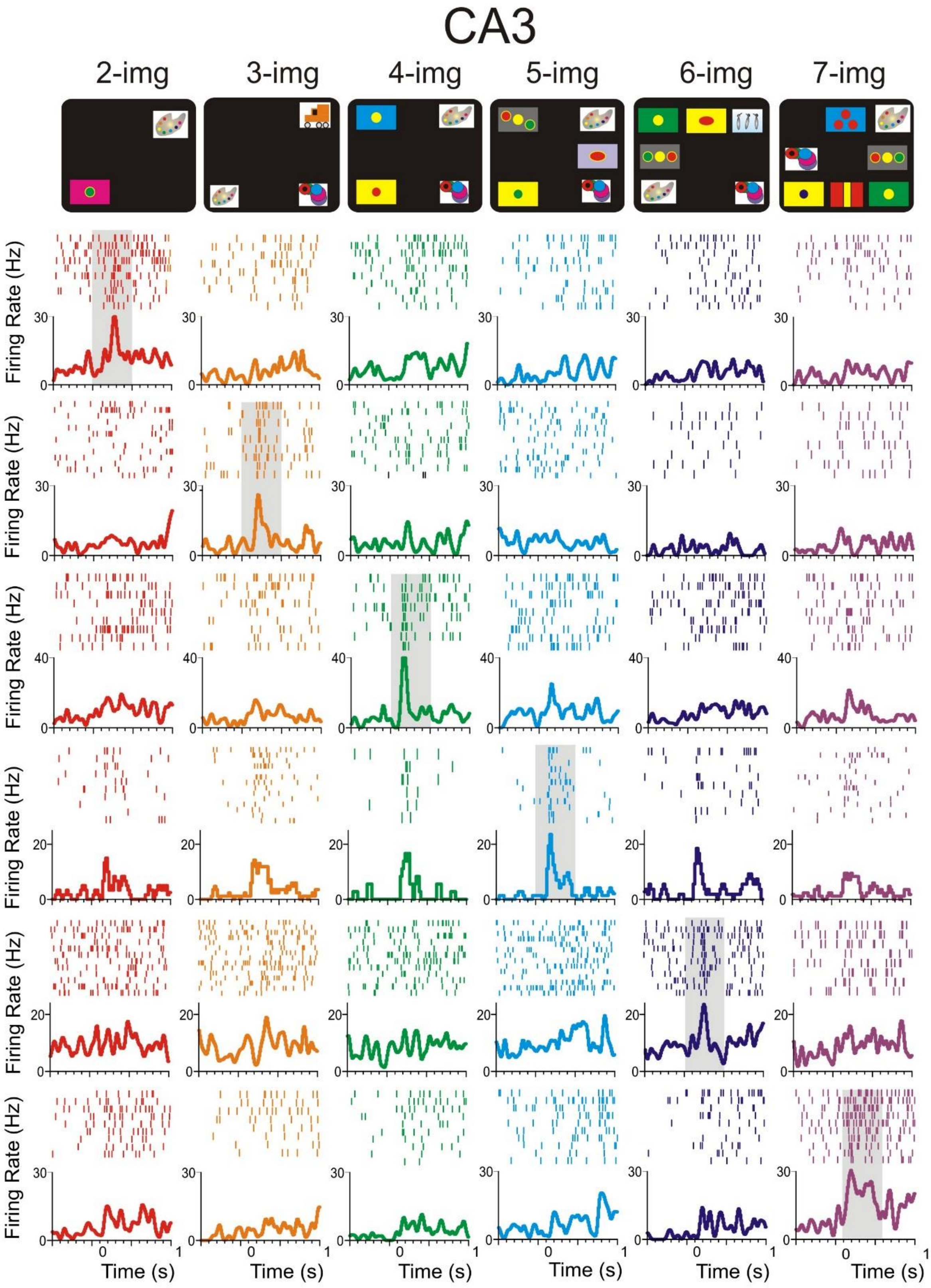

CA3
2-img
3-img
4-img
5-img
6-img
7-img
Firing Rate (Hz)
30
0
40
20
0
1
Time (s)

Figure 3S CA3 neurons firing during numeric categorization. Rasters and peri-event histograms arrays for six different CA3 cells illustrates peak firing for the selective numeric categorization of the screen in terms of number of images, similar to that shown in CA1 (Fig 2S) during the Match phase at the time of the MR (0.0s).

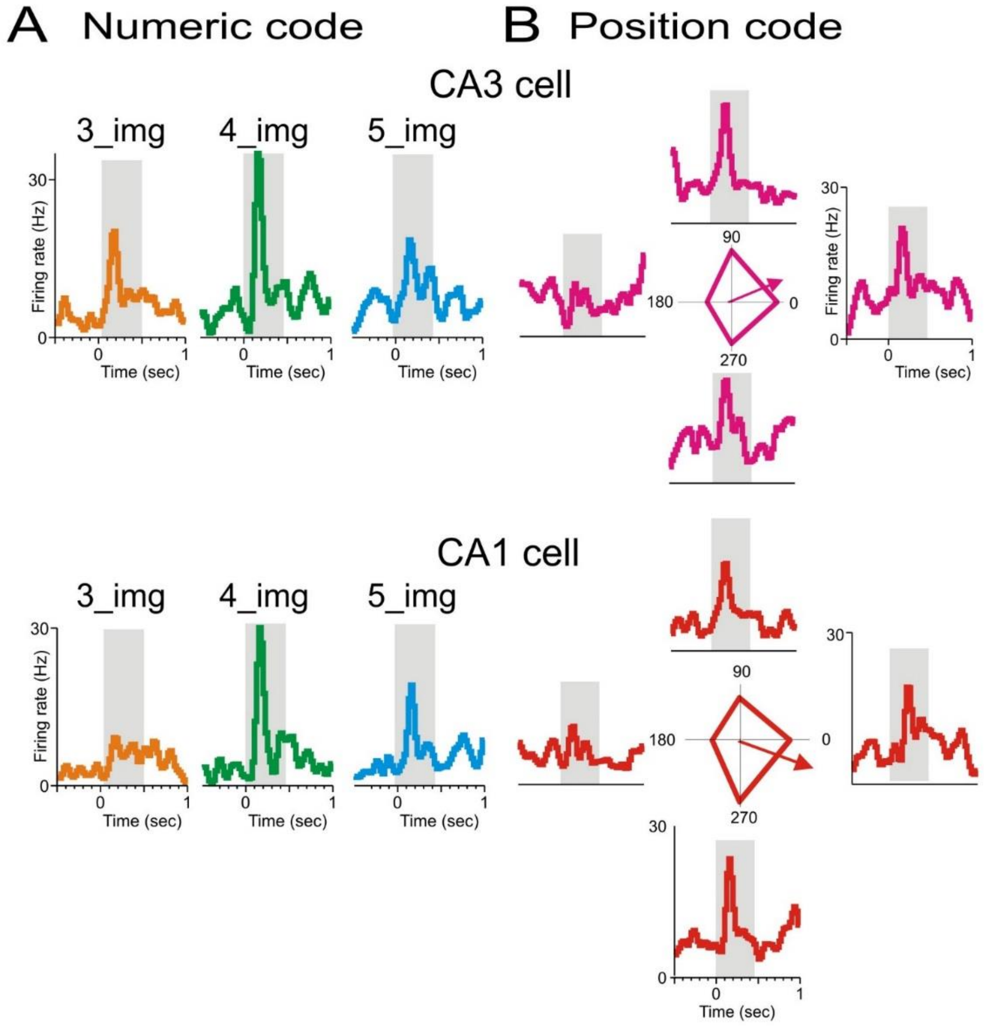

Figure 4S Distributed coding of spatial and numeric features. Comparison of numeric coding (**A**) and spatial tuning (**B**) of the MR for two different (CA1 and CA3) hippocampal cells. Image categorization and position encoding on Object and Spatial trials respectively illustrates similar processing but different sensitivities to the same features.

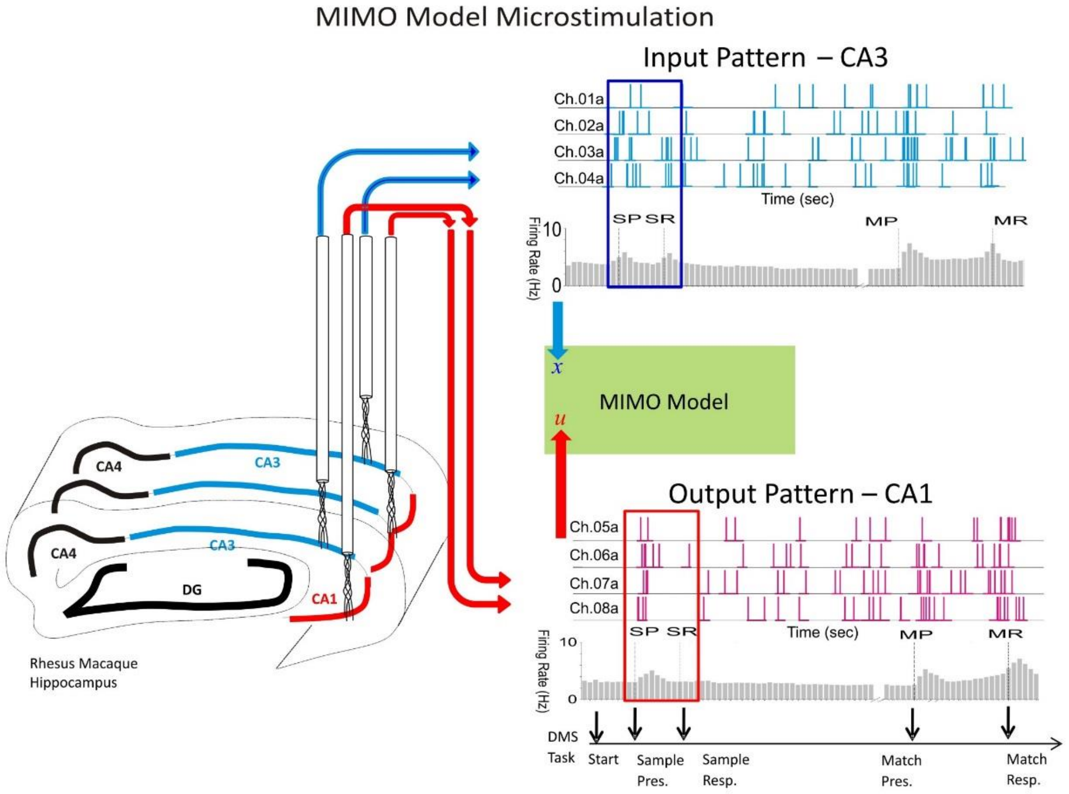

Figure 5S Schematic description of recording and stimulation in hippocampal subfields CA1-CA3. Application of previously employed multi-input multi-output (MIMO) nonlinear model (Hampson et al 2012) combined with the conformal MEA probes employed to extract the pattern of electrical (bipolar) stimulation pulses (20 uA and 1 ms) delivered to hippocampal subfield CA1 following assessment of the input pattern in simultaneously recorded CA3 neurons.

Supplementary References

55. Opris I, et al., Closing the loop in primate prefrontal cortex: Inter-laminar processing. *Front. Neural Circuits*. **6**, 88 (2012).

56. Opris I. Hampson RE, Gerhardt GA, Berger TW, Deadwyler SA Columnar processing in primate prefrontal cortex: Evidence for executive control microcircuits. *J. Cogn. Neuro* **24**, 2334-47 (2011).

57.Opris I., et al., Neural Activity of Frontal Cortical Layers: Evidence for Columnar Sensorimotor Processing. *J. Cogn. Neurosci*. **23**, 1507-1521 (2011).

58 Opris I, et al. Prefrontal cortical microcircuits bind perception to executive control. *Sci Rep* **3**, 2285 (2013).

59. Paxinos G, Huang X F, Toga AW "The Rhesus Monkey Brain in Stereotaxic Coordinates" Academic Press (2000).

60. Hampson, R.E., et al. Facilitation and Restoration of Cognitive Function in Primate Prefrontal Cortex by a Neuroprosthesis that Utilizes Minicolumn-Specific Neural Firing. *J Neural Eng*, **9**, 056012 (2012a).

61. Opris I, Hampson RE, Deadwyler SA The encoding of cocaine vs. natural rewards in the striatum of nonhuman primates: categories with different activations. *Neuroscience.* **163**, 40-54 (2009).

62. Opris I, Barborica A, Ferrera VP Effects of Electrical Microstimulation in Monkey Frontal Eye Field on Saccades to Remembered Targets. *Vision Research* **45**, 3414-3429, (2005).

63. Rao, S.G., Williams, G.V. & Goldman-Rakic, P.S. Isodirectional tuning of adjacent interneurons and pyramidal cells during working memory: evidence for microcolumnar organization in PFC.. *J. Neurophysiol*. **81**, 1903-1916 (1999).

64. Berger TW, Hampson RE, Song D, Goonawardena A, Marmarelis VZ, Deadwyler SA. A cortical neural prosthesis for restoring and enhancing memory. *J Neural Eng*. **8**, 046017 (2011).

65. Hampson, R.E., et al., Neural representation of cognitive processing in the prefrontal cortex of nonhuman primates. *Conf.Proc.IEEE Eng Med.Biol.Soc.* (2012b).

67. Hampson, R.E., et al. A nonlinear model for hippocampal cognitive prosthesis: memory facilitation by hippocampal ensemble stimulation. *IEEE Trans. Neural Syst. Rehabil. Eng*, **20,** 184-197 (2012c)

68. Hampson RE, Song D, Chan RH, Sweatt AJ, Riley MR, Goonawardena AV, Marmarelis VZ, Gerhardt GA, Berger TW, Deadwyler SA. Closing the loop for memory prosthesis: detecting the role of hippocampal neural ensembles using nonlinear models. *IEEE Trans Neural Syst. Rehabil Eng*. **20**, 510-25 (2012d).

69.Deguchi, Y., et al. Temporally matched subpopulations of selectively interconnected principal neurons in the hippocampus. *Nat. Neurosci.,* **14**, 495-504, (2011).

70. Klausberger, T. & Somogyi, P. Neuronal diversity and temporal dynamics: the unity of hippocampal circuit operations. *Science*, **321**, 53-57 (2008).
